# The influence of television content on advertisement: a neurophysiological study

**DOI:** 10.3389/fpsyg.2024.1266906

**Published:** 2024-02-02

**Authors:** Debora Bettiga, Giuliano Noci

**Affiliations:** Department of Management, Economics and Industrial Engineering, Politecnico di Milano, Milan, Italy

**Keywords:** halo effect, television content, media context, advertising, neurophysiological measures, media psychophysiology

## Abstract

Emotional and cognitive reactions to the media context prove impactful on advertising effectiveness. However, research on the topic remains lacking and with a profusion of mixed results regarding the role of the context in enhancing or detracting communication effectiveness. This study explores the media context-advertising relationship, by investigating the influence of television content on advertisement in light of media psychophysiology and grounding on the Halo effect theory. Consumers’ responses to different television content and advertisements are assessed. Specifically, consumers’ arousal, pleasure, attention, and memorization are measured through brain analysis, heart rate, and skin conductance detection. Self-reported methods complement such analysis, by exploring the values associated with the television content and the advertised brands. Results show that television content influences consumer responses to the advertisement and the values associated with the brands, confirming the existence of a halo effect. Responses differ among television content typologies.

## 1 Introduction

The mixed results coming from television advertising, being more fragmented, and the rising competition among brands spurs the advertising industry to get new insights into how customers perceive and react to promotional messages on different television channels and contexts. Gaining consumers’ attention, building memory and favorable perceptions of the brand are more and more challenging objectives in the current highly competitive environment (Pieters et al., 2002; Park et al., 2021). Despite the decrease in television attractiveness as a medium where to display advertisements, in favor of online advertising, recent studies show that TV advertisements elicit more attention and positive emotions compared to online advertisements (Weibel et al., 2019). Hence, understanding the factors that affect consumer perceptions and reactions toward advertising may help in improving commercial placement, and consequently, brand communication effectiveness.

Most of the research on the effectiveness of advertising focuses on the quality of the communication itself or the quality of the product promoted (e.g., Malthouse et al., 2007). Different studies focus on advertising creativity, but with results that are hardly generalizable to different settings (Hartnett et al., 2016). Recent evolutions in the media landscape, however, make it necessary to refocus the attention on the context in which the advertising is displayed, as a driver of effectiveness. The failure of the cookie implies that addressable advertising, which has been seen as the new advertising paradigm by companies and media makers, is not likely going to be in place anymore. This implies a return to the classical media placement using context.

Research on the context took different directions. A classic view posits that ads well designed for a specific target audience will work potentially in every medium (Rossiter and Percy, 1987) and some studies even do not confirm the effect of context. Context power has been mainly associated with the size of the audience it delivers (Graham and Kennedy, 2022). On the other side, affect transfer theory (Mattes and Cantor, 1982) has been used as a model to explain consumer reactions to advertising in different contexts. Affect refers to the consumer linking of a branded offer. It suggests that television commercials seen after arousing movies are perceived as more effective and enjoyable than the same commercials seen in less arousing television content. However, this effect is limited in time, as the second commercial in a break gets affect transferred from the previous ad (Poncin and Derbaix, 2009) as ads elicit affective reactions to ads shown in the same break. This suggests the need to carefully order ads during a commercial break to lower the negative impact of ads with lower customer retention on other ads, to avoid consumers may switch channel. This negative externality may indeed affect the network’s revenues (Shi et al., 2022).

Prior studies show that the engagement and the congruency of the media context are factors determining communication effectiveness (Dahlén, 2005; Jeong and King, 2010; Segev et al., 2014; Davtyan and Tashchian, 2022; Zeng et al., 2022). Other studies focused on the usage, interactivity, and attitude toward the medium (Kwon et al., 2019). More recent studies focus on online contexts, especially on the role of social media influencers, investigating how the interaction between the typology of social media influencers and the argument quality determine different consumer responses to advertisement, both neurological and self-reported (Sánchez-Fernández and Jiménez-Castillo, 2021; Pozharliev et al., 2022a). Regardless of the area of focus, research agrees on the impactfulness of the emotional and cognitive reactions to the media context on advertising effectiveness (Pieters et al., 1999; Venkatraman et al., 2015; Pozharliev et al., 2017). However, research on the topic remains with a profusion of mixed results regarding the role of media context in enhancing or detracting advertising effectiveness (Kwon et al., 2019). Furthermore, the focus has mainly been on affective responses, where the affect transfer theory mainly contributes, but not on the wide spectrum of cognitive responses and consumer evaluations.

This work aims to explore the role of affective and cognitive reactions to television content in influencing reactions toward the advertisement, in light of media psychophysiology. We study the media context-advertising relationship by grounding on the Halo effect (Thorndike, 1920), a mechanism that leads consumers to form evaluations of people, products, or brands by overgeneralizing and inferring across different attribute dimensions. In the domain of media psychophysiology, our premise is that the Halo Effect becomes apparent within TV consumption, particularly in the way advertisement is connected with TV content. This occurs as individuals make judgments based on contextual information, specifically the TV show in which the advertisement is placed. Instead of assessing the product or brand in isolation, consumers utilize a cognitive shortcut, associating positive or negative aspects of the TV show with the advertised content. This implies that viewers may attribute features of the surrounding TV program to the advertisement, even when the explicit characteristics of the product or brand are not plainly demonstrated.

To test such mechanism, consumers’ responses are studied through neurophysiological methods, specifically, electroencephalogram (EEG), heart rate (ECG), and skin conductance detection, that offer unbiased access to the consumers’ brain and bodily reactions to advertising stimuli (Pozharliev et al., 2017). Furthermore, neuroscience-based methods, have demonstrated their effectiveness in detecting consumer processing and reactions to ad messages (Falk, 2010; Vecchiato et al., 2010; Falk et al., 2012; Eijlers et al., 2020; He et al., 2021b; Zhu et al., 2022). The measurement of the brain reactions and the body responses can shed light on how individuals process messages (Cacioppo et al., 2007). In marketing communication, the neuroscience approach allows higher inferential traction and more reliable measurement than traditional self-reported measures (Sánchez-Fernández et al., 2021; Pozharliev et al., 2022b). Moreover, some divergent results in terms of media context-advertising effects have been attributed to measurement issues (Moorman et al., 2012). Prior studies, indeed, assessed advertising responses, in terms of emotions, attention, involvement, and memorization, through different self-reported methods, which may be subject to biases, such as social desirability bias or common-method variance (Randall and Fernandes, 1991; MacKenzie and Podsakoff, 2012). Through neurophysiological methods, we attempt to overcome this limit, by providing an unbiased assessment of consumer reactions (He et al., 2021a). Prior studies demonstrate the potential of biometrics in the measuring of television commercial effectiveness (Bellman et al., 2017, 2019). Above neuroscience-based methods, a survey is adopted to assess consumer evaluations, namely television content perceived quality, and values attached to the television contents and the advertised brands.

## 2 Halo effect in advertising

Forming accurate impressions about people and things is a very complex and demanding cognitive task, subject to several shortcuts and biases. Since reality is way too complex for a complete understanding, extrapolating from known information to unknown details is one of the most common heuristics people use (Asch, 1946; Crano, 1977). Thanks to this and other compensating effects, our brain is capable of generating a first impression after only 100-ms exposure to a human face (Willis and Todorov, 2006). Defined “Halo” (Thorndike, 1920), this instinctive heuristic has been later defined as the excess of correlation over and above the true correlation between attributes (Murphy and Jako, 1989). It assimilates the evaluation of different attributes, flattens the overall profile of evaluation, and compresses the differences among attribute evaluations (Murphy et al., 1993).

Despite its importance in the formation of consumer evaluations, few studies have been conducted on the halo effect in the advertisement field. Thanks to the halo effect, commercials placed inside programs judged as interesting and pleasant were better evaluated (Krugman, 1983). Further studies addressed the impact of website credibility on Click Through Rate, to account for the number of clicks on a link or banner over all the visualizations (Colbert et al., 2014). They confirmed the presence of a halo effect where increased credibility improves both Click Through Rate and user experience, working through perceived credibility and greater engagement. In the television field, Babad (2005) explored the halo effect of a television interviewer’s preferential behavior on the viewer’s perceptions of the interviewee. In the digital publishing sector, ads on premium publisher sites can deliver substantially greater branding effectiveness for online display ads, being thus able to support a higher Cost Per Mille (Lipsman, 2016). These studies provided the first evidence of the existence of a halo effect from the publisher to the advertisement. Further studies explored the relationship between media context and advertisement, mostly concentrating on the medium, in terms of usage, interactivity, or attitude toward it (Olney et al., 1991; Stipp, 2018; Sreejesh et al., 2021). In the context of social media communication, a congruency effect of advertising appeal and advertising channel on advertising effectiveness has been individuated (Zeng et al., 2022). Davtyan and Tashchian (2022) explored the effects of thematically congruent and incongruent brand placements, showing that while incongruent brand placements enhance consumers’ brand memory, congruent placements generate positive brand attitudes, but both exert similar effects with a high frequency of brand placement repetition. Other studies investigated more experiential aspects, such as involvement with the media and mood states (Kwon et al., 2019).

The health halo effect in food advertising has been the subject of several studies (e.g., Fernández-Escobar et al., 2021). Here, the halo effect emerges in the positive or negative impact of health messages on food choices. For instance, nutritionally lacking foods advertised with implicit and explicit references to health may lead to judgment errors about healthy food choices by misleading the understanding of nutritional characteristics (Whalen et al., 2018). Additional works confirm the halo effect generated by corporate reputation on company products in the context of television choices, where consumers are less likely to purchase products from companies with poorer reputations (Burke et al., 2018). Hence, placing products in a specific context (e.g., the context of a healthy lifestyle) within television advertising may generate a better evaluation of a product even attaching to it qualities which it does not possess and that were not claimed. Overall, the relationship between the media environment and advertising effectiveness has shown mainly positive with a favorable spill-over from the medium to the commercial (Khouaja and Bouslama, 2011).

Grounding on prior studies, we assume that the halo effect mechanism manifests in TV advertisement, by letting individuals form judgments based on contextual information, i.e., the TV show in which the advertising message is placed. Rather than evaluating the product or the brand advertised independently, viewers apply a cognitive shortcut by attaching positive or negative aspects of a TV show to the advertising, even if the product or brand attributes are not explicitly demonstrated. Thus, the halo effect impacts the cognitive elaboration of the advertisement by letting individuals form quick judgments grounded on limited contextual information. Moreover, we posit that the halo effect can generate implicit associations for specific features of a product or brand which can trigger emotional responses even when individuals are not consciously evaluating the associated product features. We delve more into this aspect in the further discussion about the link between media psychophysiology and the halo effect.

## 3 Halo effect and media psychophysiology

Mental experiences, which include advertising exposure, emerge from the ongoing activity of the brain. As clarified by the Embodied Motivated Cognition (EMC; Potter and Bolls, 2012), the brain produces a continuous stream of mental experiences at different levels of unconsciousness. As the brain is connected with the rest of the body, the peripheral nervous system assessment provides critical insights into the mechanisms underlying individuals’ exposure to communication (Potter and Bolls, 2020). Psychophysiological measures emerge as powerful indicators in media processes and effects research, being connected with variation in attention, arousal, and positive/negative emotional responses (Bolls et al., 2019). Research in psychophysiology confirms the influence of the media context on the advertisement. Following media psychophysiology, changes constantly occur when individuals interact with media, unfolding through the functioning of dynamic embodied mental processes evoked through media consumption, identified as media effects (Bolls et al., 2019). For instance, some advertising elements or specific moments in TV ads may elicit emotional responses or engagement at the physiological level, contributing to the overall Halo Effect. Such mechanism is confirmed by the Theory of Excitation Transfer, which posits, with specific regards to arousal, that consecutive dependencies exist in emotional reactions, where preceding emotion-arousing situations intensify emotions in present situations, lingering beyond the immediate exposure to that stimulus (Zillmann, 2008; Cummins, 2017). The theory treats excitation as the key driver of the emotional experience, coming from the dominance of the sympathetic activity in the autonomic nervous system (Zillmann, 1996). Following the psychophysiological paradigm, assuming that the human mind is embodied, so that all forms of human mental activity exist in the brain and are observable through neurophysiological manifestations (Bolls et al., 2019), we do assume that manifestations of the halo effect should be evident at the physiological level as well.

Prior studies confirmed such a mechanism. Clark et al. (2018) for instance, analyzed how users cognitively process advertisements embedded in mobile content, finding, among others, that users allocate more cognitive resources (assessed through heart rate change) to the ads when these were smoothly placed within the content. Hence, the halo effect manifests at the psychophysiological level, where positive affect, arousal or cognitive engagement toward the television context may lead to positive emotions, arousal, attention or memorization during the advertisement exposure. Thus, the halo effect impacts cognitive mechanisms by simplifying decision-making through heuristic processing. It also influences emotional processes by creating emotional contamination, through implicit associations and neurophysiological reactions.

Advertising research suggests three key indicators of message effectiveness: attention, memorization, and emotional engagement (Pieters et al., 1999; Langleben et al., 2009; Vecchiato et al., 2010, 2012; Venkatraman et al., 2015; Pozharliev et al., 2017) that may be subject to such halo effect. Emotional engagement, indeed, may produce a halo effect for positive beliefs, while reducing halo for negative beliefs (Bagozzi, 1996). Emotional reactions can also create a halo effect which influences the entire memorization process, by allowing a more in-depth analysis of the advertisement’s features by the individual (Lombardot, 2007). Here, the first reaction to an advertisement can have a positive influence on attention toward the message and facilitate the emergence of a halo effect, a general first impression which is easily accessible in the memory and which allows the individual to remember other advertisement elements (Srull and Wyer, 1989; Lombardot, 2007).

The methodological aspect is also worth noticing. Indeed, prior studies tested the halo effect mainly by adopting self-administered surveys, looking for changes in participants’ attitudes. However, the halo effect is a mechanism leading to unconscious alteration of judgments (Nisbett and Wilson, 1977) when an initial positive judgment about an object unconsciously colors the perception of the object as a whole. Thus, neurophysiological methods, by providing an unbiased assessment of consumer reactions (He et al., 2021a) seem a more desirable method to assess such mechanisms. By depicting peaks in emotional intensity, moments of heightened arousal or brain activity associated with different stages of content processing, such as attention or memorization, it is possible to understand which aspects of a content contribute to a certain cognitive or emotional response which can be transferred to the ad. Plus, neurophysiological methods, being real-time measurements, enable us to assess transitions between positive and negative emotional states and to measure punctual emotions or reactions, depicting how the halo effect transfer evolves over time. For instance, skin conductance, by assessing changes in arousal, reflects the emotional engagement of a consumer. A heightened skin conductance, thus, indicates emotionally impactful moments in the content which can contribute to the overall positive or negative evaluation of the television content and influence subsequent evaluations of the advertisement message attributes. Hence, neuroscience can support in measuring how specific response patterns of nervous system activity are indicators of punctual mental processes (Bolls et al., 2019).

Physiological markers help pinpoint specific ad elements or sequences that trigger emotional and cognitive responses in real-time. These methods prove valid to measure television commercial effectiveness (Bellman et al., 2017, 2019) and offer unbiased access to the consumers’ brain and bodily reactions to advertising stimuli (Pozharliev et al., 2017). Neuroscience-based methods, indeed, have demonstrated their effectiveness in detecting consumer processing and reactions to ad messages (Falk, 2010; Vecchiato et al., 2010; Falk et al., 2012; Eijlers et al., 2020; He et al., 2021b; Zhu et al., 2022) and allows higher inferential traction and more reliable measurement than traditional self-reported measures (Sánchez-Fernández et al., 2021; Pozharliev et al., 2022b). To that, we should add that self-reported measures may be subject to biases such as social desirability bias or common-method variance (Randall and Fernandes, 1991; MacKenzie and Podsakoff, 2012). Some of the divergent results in terms of media context-advertising effects could be indeed attributed to such measurement issues (Moorman et al., 2012). Furthermore, values embedded in programs and advertisements represent the drivers of interest in consumers (Pollay, 1983; Czarnecka et al., 2018) where the selection of a medium that indirectly communicates the brand values may increase ad effectiveness (De Pelsmacker et al., 2002; Dahlén, 2005). In the following, attention, memorization, emotions, and value perceptions are deepened.

### 3.1 Attention

Attentional mechanisms determine which information the individual elaborates on and which he ignores, having a remarkable role in decision-making (Pozharliev et al., 2015; Shaw and Bagozzi, 2018). Due to the massive amount of incoming commercial information and the limited processing capacity of individuals, the attention a message can gain has become a crucial indicator of advertising effectiveness (Shaw and Bagozzi, 2018; Simmonds et al., 2020). Generating attention from consumers is the first fundamental step of the purchasing process, both for new products and for familiar ones, for instance by nudging memory.

The attention devoted to television content is likely to spill over to the advertisements, hence resulting in greater effectiveness (Moorman et al., 2012). The transfer hypothesis predicts that context-induced involvement is transferred to the advertisements that follow in the commercial break in a sort of spill-over effect (Krugman, 1983). For instance, in magazines, absorbing content can generate positive reactions to advertising messages (Malthouse et al., 2007). Similarly, on websites, consumer involvement with the media context positively affects advertising effectiveness (Calder et al., 2009). In the television context, attention given to a program is been kept active during the subsequent commercial (Krugman, 1983). Conversely, negative experiences appear not to damage the advertising effectiveness in magazines, potentially because of the freedom to choose the content, contrary to what happens in linear television (Malthouse et al., 2007). Grounding on this evidence, we assume that attention toward the television content may influence attention toward the advertisement, both in positive and negative ways. More formally:

*H*1: Greater (lower) attention toward the television content will increase (decrease) attention toward the commercial

### 3.2 Memorization

Memory has been described as “any physical change that carries information about the historical past” (Redish and Mizumori, 2015). Memorization is the process of encoding, consolidating, and retrieving information. It plays an essential role in the learning and decision-making process, enabling recall and recognition of brands, which are fundamental indicators of advertising effectiveness (Jun et al., 2003). Prior research demonstrates that the specific context in which the advertisement is placed could strongly impact its memorization. For instance, individuals exposed to the advertisement in a specialty magazine were less able to discriminate the product advertised from other products of the same category than those exposed to a general audience magazine (Jun et al., 2003). Memorization shows to increase when consistency between the advertisement and the television context is assured (Simola et al., 2013), both in print (Moorman et al., 2002) and online (Hervet et al., 2011). However, prior studies found divergent results on the relationship between media context and ad memorization, with both positive and negative impacts (Moorman et al., 2012). Such mixed results may be due to the measurement itself of memorization, sometimes operationalized as recall, others as recognition of the stimulus (Moorman et al., 2012). Moving from that, we assume that a transfer mechanism may be in place for television advertisements, where the memorization of the television content could affect the memorization of the commercial. More specifically:

*H*2: Greater (lower) memorization of a television content will increase (decrease) memorization of the commercial

### 3.3 Emotions

Emotions, identified in the two dimensions of pleasure and arousal, constitute relevant, predictable, and impactful drivers of decision-making and post-decision appraisal (Ekman, 1992; Lerner et al., 2015; Bettiga et al., 2020). Pleasure (or valence) reflects happiness and delight while arousal conveys excitement, stimulation, and bodily activation. Arousal is a fundamental component of behavior (Groeppel-Klein, 2005). It indicates an active body reaction to relevant outside stimuli and their processing and is a driver of decision-making processes (Groeppel-Klein, 2005; Bettiga et al., 2017). Arousal can have a positive or a negative valence: for instance, a subject highly aroused may be both positively excited while watching an action movie or scared by a horror movie. On the opposite, low arousal could indicate both relaxation, such as what may happen when watching a pleasant television program, or boredom, while watching a monotonous one. This view has been widely confirmed through empirical studies (Baker et al., 1992; Ward and Barnes, 2001) that revealed the divergent form of the arousal-relaxed and pleased-unpleased dichotomy.

The emotional appeal of the context has been shown to moderate the responses to advertisements (Janssens and De Pelsmacker, 2005). Arousal has repeatedly been displayed to influence reactions to advertising messages (Eijlers et al., 2020; Jiang et al., 2020). In terms of valence, humorous advertisements are shown to be more effective when placed in positive mood programs and breaks (Khandeparkar and Abhishek., 2017). Murry et al. (1992) by manipulating emotions, showed that if an individual likes a program, he would equally like the following commercial, confirming that feelings elicited by television content determine the consumer evaluation of adv. Affect and mood induced by the media content are carried over on advertisement evaluation (Krugman, 1983; Frarice and Whaii Park, 1997). For instance, liking a program puts consumers in a positive mood, which is likely to be transferred to the commercial following the program (De Pelsmacker et al., 2002). The effects of pleasure and arousal, indeed, decay quite slowly, extending their influence from the program to the commercial (Vanden Abeele and MacLachlan, 1994). More recently, Breuer et al. (2021) showed that low-to-moderate arousal and valence-neutral responses improve the attention toward sponsor messages during a live sports broadcast. Grounding on this discussion, we hypothesize that the emotional appeal of the television content, in terms of valence and arousal, will impact emotional responses toward the commercial. More specifically, we propose that:

*H*3: Greater (lower) arousal toward a television content will increase (decrease) arousal toward the commercial which follows

*H*4: Greater (lower) pleasure toward a television content will increase (decrease) pleasure toward the commercial which follows

### 3.4 Value perceptions

Television programs and advertisements transmit values to the public (Cheng and Schweitzer, 1996; Lin, 2001). Values represent cultural principles, meanings, and symbols of which national culture is an important determinant (Czarnecka et al., 2018) and they may impact learning (Samaniego and Pascual, 2007). News, for instance, displays frequency, meaningfulness simplicity, and consistency values (Greguš and Mináriková, 2016). The usage of mass media on the reverse has been associated with materialistic values (Rai et al., 2020). Such values should align with the values of the target market and represent the drivers of interest in consumers (Pollay, 1983; Czarnecka et al., 2018). Theories on the media-context effect show that creative media choice (i.e., selection of a medium that indirectly communicates the message) increases ad effectiveness, in terms of brand associations, ad credibility, and brand attitudes (Dahlén, 2005). For instance, advertising messages displayed in appreciated television and print contexts determine a better attitude toward the advertisement (De Pelsmacker et al., 2002). This does not happen when the commercials are presented in disliked programs (Schumann, 1986). Bronner and Neijens (2006) further studied the relationship between media experience and how the advertisements shown in the media are experienced. They found that if a television program is judged as stimulating for the consumer, the advertising within this program is also experienced as stimulating. However, no further correlations were found for the television media. Grounding on this discussion, we assume that a transfer of values, according to the media context-advertisement effect, occurs in the television media. More formally:

*H*5: The values perceived toward television content will influence the values perceived toward the commercial

## 4 Materials and methods

### 4.1 Sample selection: stratification and pre-screening test

We conducted a laboratory experiment on an experimental base composed of 60 Italian individuals, equally distributed in gender with balanced, but different backgrounds. The subjects were selected and equally distributed in three age ranges: 21 subjects from 18 to 34 years old, 19 subjects from 35 to 49 years old, and 20 subjects from 50 to 64 years old. The sample size is in line with prior studies using biometric methods (Vecchiato et al., 2010, 2012; Vecchiato et al., 2011). Participants were selected through a pre-screening survey where we assessed consumer evaluations of the brands that will be displayed during the laboratory experiment and collected demographic information needed to stratify the sample. The pre-screening survey was conducted some days before the laboratory experiment, to further ensure that participants’ responses during the laboratory experiment are not biased by prior responses to the pre-screening survey. The pre-screening test presented five additional brands above the six to be used in the study, to avoid distortion in brand recall during the laboratory experiment, as participants would not easily connect the brands displayed in the commercial with the ones evaluated during the pre-screening survey The brand evaluation assessment was conducted by using the 3-item scale proposed by Chandon (2003). This served to verify that the brand’s evaluation was not deeply negative, to avoid distortions in experimental results. Familiar, favorable brands, indeed, have been shown to elicit different neural responses than familiar but unfavorable brands (Esch et al., 2012).

### 4.2 Stimuli selection

Six television advertisements promoting different brands and six different television shows were tested. Such a number ensures from one side the generalizability of our results to a broad range of adv, from the other side picking a limited number allows the experiment to be conducted in a reasonable time, to avoid participants feeling bored or stressed by the length of the testing. The advertising messages were chosen by a team of four experts operating in the advertising field. Each advertisement was 30 s long. They displayed consumer good brands, familiar to the population from which participants have been selected, and with a wide target group. Television shows pertain to three common typologies: TV series (a series of episodes created or adapted for television broadcast and related to a subject), entertainment shows (entertained aimed music-recreation, game and quiz shows, talk and variety), and reality shows (television program documenting how people behave in everyday life or in specific situations created by the program maker). This is to ensure that different television contexts are considered, due to their specificities and potential differential impact on the relations under test. For each program typology, one high-quality and one low-quality television content were selected, for a total of six television shows under test. The quality of the television content, indeed, may affect the relationship between media content and advertisement (Gunter et al., 1997) where specific editorial quality may impact advertising success (Sommer and Marty, 2015). The level of quality was defined by a team of four experts operating in the advertisement field. We further verified the validity of such classification by asking participants to rate each television show’s quality through a 5-point Likert scale, outcomes are shown in the results session. Overall, the following television program typologies were displayed: high-quality TV series, low-quality TV series, high-quality entertainment shows, low-quality entertainment shows, high-quality reality shows, and low-quality reality shows.

### 4.3 Neurophysiological measures

Neurophysiological indicators, indices of bodily responses reflecting changes in physiological responses (Potter and Bolls, 2012) of the subjects’ reactions, were measured during the experiment using biometric techniques. This choice has been made as both cognitive and affective reactions of consumers are hardly measurable through self-reported methods, being individuals usually not able to correctly assess and report the emotions they experience and their cognitive effort (Chamberlain and Broderick, 2007). Three types of biometric measures have been recorded, following physiopsychological research which suggests the need for multiple measures (Cacioppo et al., 2016): electroencephalography (EEG), electrocardiography (ECG), and skin conductance (SC) signal. EEG signal allows the assessment of the brain activity of the participants, while ECG and SC signals permit to measure the autonomic nervous system activation. In the advertising field, heart rate is used as a psychophysiological indicator of cognitive resource allocation (Clark et al., 2018). Skin conductance has been validated as a measure of arousal, as well as anxiety, in response to different media contexts (Bolls et al., 2019). EEG has been adopted to study multiple forms of cognitive and emotional processes in media consumption (Morey, 2018). By recording electrical signals generated by the firing of neurons in activated cortical areas, cortical activity can be recorded from both hemispheres allowing inferences about mental processes engaged during various tasks, including media exposure (Bolls et al., 2019). Using different and complementary measurements permits a comprehensive appraisal of the individual reactions to the television show and the advertising message.

#### 4.3.1 Electroencephalography measures

The EEG was acquired using a portable 64-channel system (SD LTM Express and System Plus Evolution software, Micromed, Italy). To guarantee easy and fast use of this measuring system, we employed 27 electrodes over the 64 available, that were uniformly distributed on the scalp to cover all the most relevant activation regions of the brain, that are the frontal, central and occipital regions. Each electrode was filled with a water-based gel to enhance conductivity with the participants’ scalp. The EEG activity was collected at a sampling rate of 128 Hz and the impedance level was kept below 5 kΩ for all the acquired electrodes. Three indexes were calculated from the brain activity signal: Memorization Index (MI), Attention Index (AI), and Pleasure Index (PI; Chaouachi et al., 2010; Vecchiato et al., 2010, 2012). Each one of these indexes was obtained by computing the Global Field Power (GFP) of the EEG signal in a specific frequency band. The bands were related to the Individual Alpha Frequency (IAF) of the subject (Chaouachi et al., 2010; Vecchiato et al., 2010, 2012). Specifically, MI was calculated from the frontal electrodes (F3, AF3) activation in theta band = [IAF-6, IAF-4]. AI was calculated from the frontal electrodes (F3, AF3, F4, AF4, Fz, FPz) activation in the low alpha band = [IAF-4, IAF]. PI was calculated (Equation 1) separately from the left (F3, AF3, F1) and right (F4, AF4, Fp2) electrodes activation in high alpha band = [IAF, IAF + 2] and expressed as:

(1) Pleasure=Pleasure:left−Pleasure:right

All the indexes were obtained by temporarily averaging their values during the vision of each content of the experiment (television program and advertisement). The mean values thus obtained were related to the mean value recorded during the vision of a neutral image (baseline) and reported on a percentage scale using the following formulas:

(2) $$\begin{array}{l} \mathrm{Index advertisement}=\\ \left(\mu \_\mathrm{adevrtisement}-\mu \_\mathrm{baseline}\right)/\mu \_\mathrm{baseline}\times 100 \end{array}$$

3) $$\begin{array}{l} \mathrm{Index TVprogram}=\\ \left(\mu \_\mathrm{TVprogram}-\mu \_\mathrm{baseline}\right)/\mu \_\mathrm{baseline}\times 100 \end{array}$$(

#### 4.3.2 Skin conductance and electrocardiography measures

A galvanic skin response sensor has been used to measure the electrodermal activity (EDA) of the participants, with electrodes placed on the individual’s fingers through a band. The SC signal provides a measure of the electrodermal activity that is related to the skin resistance’s variation. The EDA is related to the skin resistance’s variation due to the sweating that is controlled by the sympathetic nervous system (SNS) and it increases linearly with a person’s level of physiological and psychological arousal. Waves have been measured at small intervals up to 10,000 times per second (Morin, 2011).

ECG measurement has been used to record the subjects’ heart activity and to derive the heart rate (HR), that is the speed of the heartbeat measured by the number of contractions of the heart per minute. The link between the heart rate and the emotional state of the individual is confirmed in research (Montano et al., 2009; Fortunato and Giraldi, 2014). From the ECG signal, the temporal distance between consecutive R peaks was extracted using the Pan-Tompkins method (Pan and Tompkins, 1985) and the heart rate (HR) was then calculated by computing the reciprocal of each R-R interval.

Both skin conductance and heart rate were used as some individuals will respond to stimuli with greater changes in heart rate than in skin conductance, while others will have large increases in skin conductance but only small increases in heart rate. Within subjects, changes in SC and HR can correlate positively even though there is zero or a negative correlation between subjects (Revelle and Loftus, 1992). The ECG and SC were both acquired through the ProComp Infiniti system and Biograph Infiniti software (Thought Technology Ltd., Canada). The first signal was recorded at a sampling rate of 2048 Hz, while the other one was collected with a sampling rate of 256 Hz. The mean values of HR and SC were calculated during the vision of each content and their values were related again to the mean value obtained during the vision of the neutral image (baseline) and reported on a percentage scale (Equations 2, 3). In this way, it was possible to evaluate the variation of the emotional state of the subject, correlated to the autonomic nervous system activation, concerning the steady state (baseline).

The value associated with these parameters is a particular index computed as follows:

(4) $$\mathrm{Parameter}\;\mathrm{level}=100*\frac{\left(\begin{array}{l} \mathrm{Average}\;\mathrm{parameter}\;\mathrm{measure}\\ -\mathrm{Average}\;\mathrm{baseline}\;\mathrm{measure} \end{array}\right)}{\mathrm{Average}\;\mathrm{baseline}\;\mathrm{measure}}$$

Equation (4) represents the percentage change in the mean evaluation of the parameter for the mean value recorded during the vision of the neutral image before the beginning of each part of the experiment (the baseline).

### 4.4 Self-reported measures

Above physiological assessment, for each participant we collected (i) demographic information: age, gender, education level, job; (ii) perceived quality of the television content, in terms of trustworthiness, measured on a 5-point Likert scale (iii) values associated with the brand and with the television show, measured on a 5-points Likert scale. Specifically, the following values were assessed: familiar, formative, innovative, intercultural, sustainable, esthetic, original, dynamic, and funny. These have been identified by the team of experts as the commonly assessed values in television programs and advertising.

### 4.5 Experimental flow

The experiment was conducted inside a university neuroscience laboratory. The experiment follows a between-subjects design. The experimental flow is depicted in Table 1. One participant per time took part in the experiment, to avoid the interaction with other individuals and further elements of interference. The experimental flow was the following: once the participant has been welcomed to the laboratory, he/she signs a consent form that illustrates the tools used during the experiment and the task required. Subjects have the right to withdraw at any moment from the testing. Each participant then fills out a questionnaire aimed at collecting demographic and psychographic information. Following this, each subject was equipped with an electroencephalography (EEG), electrocardiography (ECG), and a skin conductance (SC) device. All the stimuli were displayed on a computer. The first part of the testing consists of watching a neutral image. This had the objective of making people feel more comfortable and relaxed and allowed researchers to observe which were the subject’s base parameters against which to compare the reactions to the stimuli. The assessment of such baseline permits to depurate from individual variations in physiological state, thus enabling a comparison among individuals. Secondly, it depurates from potential variations in individual physiological states due to the wearing of the tools.

**Table 1:** Experimental flow.

Experimental phases	1	2	3	4	5	6	7
Group 1	Neutral image	-	Advertisement	Advertisement	-	Pause	Survey compilation
Group 2 Group 3	Neutral image	TV show	Advertisement	Advertisement	TV show	Pause	Survey compilation
Time needed	30 s	3 min	30 s	30 s	1 min	1 min	3 min

The 60 participants were then divided into three groups demographically balanced (group 1, group 2, and group 3). Group 1 only watched commercials, to record the reactions generated by the advertisement itself, without contextualization. Exposure of Group 1 to the advertising messages had the objective of evaluating if significant differences existed among the advertising campaigns. This is needed to ensure that potential differences in responses to the advertisement are not due to differences among the commercials themselves. Participants of Group 2 and Group 3 saw three blocks of television content plus advertisement stimuli. Thus, each subject of Group 2 and Group 3 saw six advertisements and three television programs (one reality show, one TV series, and one entertainment show). Each block presents 3 min of the television show, followed by two commercials, followed again by 1 min of the same television show. Between the television show and the commercial, a bumper is displayed, as happens in real television consumption. The testing flow, indeed, wants to be as representative as possible of the actual television experience. All stimuli (TV shows and advertising) were randomized: individuals see the commercials along different program typologies in a randomized order. At the end of each block, after a pause of 1 min, participants were asked to fill out a questionnaire aimed at measuring value perceptions associated with the brands displayed. Groups 2 and 3 in addition reported the values associated with the television shows.

## 5 Results

### 5.1 Preliminary checks

First, we evaluated potential differences in terms of physiological responses of pleasure, attention, memorization, and arousal among the commercials displayed. An ANOVA test shows that the six advertisements had an average pleasure of −2.71 (SD 18.64) with *F*(5.349) = 0.813 (*p* > 0.05), an average memorization of 0.32 (SD 17.16) with F(5.349) = 1,218 (*p* > 0.05), an average attention of −1.73 (SD 12.6) with F(5.349) = 1.94 (*p* > 0.05), an average arousal (HR) of 1.18 (SD 5.60) with F(5.349) = 0,907 (*p* > 0.05), and an average arousal (SC) of 8.12 (SD 20.54) with F(5.349) = 0,848 (*p* > 0.05). Hence, all measures show no significant differences among the six advertisements. Secondly, we assessed the congruency between the expert evaluation of the television content quality and the subjects’ perceptions. Analysis of variance showed a statistically significant difference at the *p* < 0.01 level in quality perception scores. Post-hoc comparisons using the Mann–Whitney test confirmed that participants evaluated the low-quality television programs (classified by experts) as significantly less trustworthy compared to the programs classified as high quality, confirming experts’ classification. This result holds also when evaluated across program typologies, age, and gender. Table 2 shows the mean values for high-quality and low-quality television programs. Further, we conducted a regression analysis to measure the potential relation among self-reported and biometric data. Results show there is no statistical significance on the coefficients for none of the parameters considered. Table 3 presents the results.

**Table 2:** Television content perceived quality.

	Groups	Value of *p*	Mean perception of high-quality content	Mean perception low-quality content
Television program typology	TV series	< 0.01	4.16	3.48
	Entertainment	<0.001	3.64	2.12
	Reality show	<0.001	2.88	1.72

**Table 3:** Results of the regression between self-reported and biometric measures of attention, memorization, pleasure and arousal.

Value	B_0_	B_1_
Pleasure	16.68	−4.79
Attention	−9.08	1.50
Arousal (SC)	3.85	−0.16
Arousal (HR)	−23.26	4.60

*value of *p* < 0.05; **value of *p* < 0.01; ***value of *p* < 0.001.

### 5.2 Hypotheses testing

We evaluated the transfer of consumer neurophysiological reactions (pleasure, arousal, attention, memorization) from the television content to the advertising messages displayed inside the television show. We measured such transfer by regressing the consumer reactions measured while watching the television program with the reactions measured during the commercial, computed by television program typology. Results show a strong linear dependence between these two measurements for all the parameters (Table 4). This suggests that a transfer effect is in act: if individuals are highly aroused during the view of the program, they also are highly aroused during the view of the advertisement; if they feel pleased by the television content they also feel as such during the commercial; the same holds for attention and memorization. The relationship is significant for each program typology. It must be pointed out that observations are distributed above and below zero and intercepts of the linear models are not statistically relevant. This fact is due to the nature of the indexes adopted (see methodology) which account for the percentage variation from the baseline. Hence, negative values must be interpreted as lower than the baseline of that specific percentage and not as negative in absolute value. Overall, the results confirm our H1, H2, H3, and H4 sustaining the relevance of the media content in determining responses toward the advertisement.

**Table 4:** Results of regression between the television content and advertisement on neurophysiological attention, memorization, pleasure, and arousal.

Television program typology	Value	B_0_	B_1_
TV series	Pleasure	−0.65	1.00***
	Memorization	1.66	1.13***
	Attention	0.98	0.97***
	Arousal (HR)	1.27*	0.87***
	Arousal (SC)	2.11	1.29***
Entertainment show	Pleasure	−1.57	0.91***
	Memorization	−3.14*	0.56***
	Attention	−3.42***	0.70***
	Arousal (HR)	1.61***	0.84***
	Arousal (SC)	−1.03	1.09***
Reality show	Pleasure	−1.80	1.05***
	Memorization	−0.35	1.08***
	Attention	−2.20***	0.99***
	Arousal (HR)	0.79	0.93***
	Arousal (SC)	2.39	1.51***

*value of *p* < 0.05; **value of *p* < 0.01; ***value of *p* < 0.001.

We further analyzed if differences exist in pleasure, attention, memorization, and arousal among participants who saw the advertisement only (Group 1) compared to the ones who saw it inside a television show (Group 2 and Group 3). Such values are the result of the difference between the parameter values recorded meanwhile the participants were watching the neutral image and while they were seeing the advertisements. Kruskal-Wallis’s one-way analysis showed no significant differences in pleasure, attention, and memorization. Conversely, results show that individuals who saw only the advertisements recorded a higher arousal level in comparison to the individuals who saw the advertisements inside a television show. Significant differences among the mean values of the groups are displayed through a Dunn-test (Bonferroni adjusted) where a higher arousal level (M = −0.05) is displayed for the group who only watched the advertisements in comparison to the subjects who saw the advertisements inside a television show, either a high-quality one (M = −10.63) or a low-quality one (M = −8.77).

Finally, we tested the existence of a transfer effect between the values associated with the television program and the values associated with the advertised brands. We performed a correlation between program evaluation and brand evaluation on each value: familiar, formative, innovative, intercultural, sustainable, esthetic, original, dynamic, and funny. Both TV series and reality shows scored statistically significant on 7 out of 9 values, meaning that a transfer effect can be found in these values. For entertainment programs instead, we encountered this effect on two values only. Overall, our results partially confirmed H5 as individuals tend to attach to brands advertised an evaluation that is consistent with the one provided for programs, even if this is not consistent among all TV program typologies. Table 5 shows the results.

**Table 5:** Correlation between value perceptions of the television content and the advertised brands.

Value	TV series	Entertainment show	Reality show
Familiar	0.12	0.06	0.28**
Educational	0.26**	−0.01	0.23*
Innovative	0.39***	0.20*	0.24*
Intercultural	0.18	−0.07	0.22*
Sustainable	0.28**	0.11	0.38***
Esthetic	0.26**	0.14	0.26**
Original	0.42***	0.12	0.23*
Dynamic	0.25*	0.07	0.13
Funny	0.35***	0.11	0.19

*value of *p* < 0.05; **value of *p* < 0.01; ***value of *p* < 0.001.

## 6 Discussion

The study investigates the media context-advertising relationship, by examining the influence of television content on television advertisement, grounding on the Halo effect theory. A halo effect between consumer reactions toward the television content and the advertisement emerges. Findings show that such an effect is relevant for all the key metrics of advertising effectiveness, namely attention, memorization, pleasure, and arousal, quantified through neurophysiological assessment. Although previous studies found divergent results in terms of media context-advertising relation (Khouaja and Bouslama, 2011; Davtyan and Tashchian, 2022), findings reveal that a strong positive relationship occurs between the two, confirming the existence of a halo effect from the television content to the commercial. We thus support, through neurophysiological assessment, prior studies which hypothesize a spill-over effect from the media context to the advertisement that follows in the commercial break (Krugman, 1983; Frarice and Whaii Park, 1997; Moorman et al., 2012). On the other side, we show that negative experiences, in terms of low attention, memorization, or emotional activation, negatively impact advertisement consumption, by hindering attention, memorization, and emotional experience, contrary to prior findings on the media context-advertisement relationship (Malthouse et al., 2007). Hence, the halo effect works both on positive and negative sides.

It is interesting to notice that consumers tend to experience higher arousal when they watch commercials without contextualization than when these are placed inside television shows. This is confirmed for both high-quality and low-quality programs. When consumers are highly involved with television content, indeed, they concentrate on the source of their arousal and devote fewer resources to commercials that interrupt the program (Newell et al., 2001). The advertisement exposure could represent a sort of break from television consumption with a consequential decrease in arousal. Thus, when the advertisement is displayed alone, it can generate greater involvement from the consumer.

Further on, findings partially support the hypothesis that value perceptions are transferred from the program to the advertised brands. For instance, brands advertised inside innovative television programs are perceived as more innovative as well. Such effect is confirmed mainly for reality shows and TV series, but only to a reduced extent for entertainment programs. The specific results for entertainment shows may derive from the peculiar characteristics of these programs (Besley, 2006). Overall, findings support the assumption that brand evaluation is shaped by the context, above the offering itself. This is true for both low-quality and high-quality content.

## 7 Academic implications

Findings from this study could help researchers to disclose new insights into the impact of the medium on the affective and cognitive processing of an advertising message. First of all, this work proves the existence of a halo effect from the media content to the advertisement on attention, memorization, pleasure, and arousal which, measured through physiological assessment, shift from the media context to the advertisement. Furthermore, such an effect exists also on value perceptions, with spillovers from the media content to the brand, moderated by the program typology. These findings contribute to research on the media context-advertising relationship by showing the existence of a halo effect between the media content and the advertisement message. Despite the importance of the halo effect in the formation of consumer evaluations, indeed, to the best of our knowledge, such a relationship has not been depicted before. An abundance of diverse results exists regarding the role of media context in enhancing or detracting from advertising effectiveness (Kwon et al., 2019). Our study helps fill this gap in research by investigating the presence of a halo effect, not only in terms of value perceptions but also expressed through physiological responses. We employed neurophysiological methods to assess the transfer of cognitive and affective reactions, specifically electroencephalogram (EEG), heart rate (ECG), and skin conductance detection, differently from prior studies grounded on self-reported measures.

Psychophysiological reactions emerged as key indicators of advertising processing, being connected with variation in attention, memorization and emotional responses, supporting prior research (Bolls et al., 2019). The findings of the study confirmed the influence of the media context on the advertisement, supporting prior research on media psychophysiology (Bolls et al., 2019). Thus, we enrich prior research on arousal as a mechanism to induce the halo effect (Bagozzi, 1996), by showing that emotional engagement with the consumption context may produce a halo effect on the advertisement. Furthermore, we showed that attention and memorization toward the television context transfer to the advertising messages placed inside that context. Prior research showed that the first reaction to an advertisement can have a positive influence on attention and memorization (Srull and Wyer, 1989; Lombardot, 2007). We further demonstrate that such a mechanism is in place also between the television context and the advertisement.

The value of adopting neurophysiological measures resides also in their ability to assess the cognitive and affective reactions of consumers, which are hardly measurable through self-reported methods, as individuals are usually not able to identify and report emotions or cognitive effort (Chamberlain and Broderick, 2007). Such assessment offers unbiased access to consumers’ brain and bodily reactions to advertising stimuli (Pozharliev et al., 2017). Furthermore, it helps to overcome the limits of self-reported methods which could explain the divergent results in terms of media context-advertising effects found in prior research (Moorman et al., 2012; Sánchez-Fernández et al., 2021; Pozharliev et al., 2022b). Hence, this work may represent a methodological reference for further research, providing directions on the use of biometric measures for the evaluation of consumer reactions and feelings toward the advertisement.

## 8 Managerial implications

This work provides several insights into how television content may influence consumer reactions toward the advertisement. We confirmed that physiological responses to TV content determine the responses to subsequent advertisements. Hence, marketers seeking new ways for creating arousal, attention, or a sense of pleasure with their advertisement, should focus, above the content itself, on the context in which the commercial is displayed. TV shows that generate attention can make consumers more attentive to the advertisements shown inside such programs. Attention is key when managing brand and product communication, as it has a direct impact on sales. Attention the individual may pay is limited and more difficult to capture, seeing the abundance of branded content available. On this metric, television is still a very powerful media, able to get active attention from consumers, which is more consistent across the ad duration than on digital media. However, the assumption that TV advertising is fully viewable is not true. Even if the full screen is devoted to the advertising message, consumers may mute the device, navigate in another screen or simply be in another room while the commercial is displayed. The same pattern seen for attention holds for memorization of the brand, where memorization toward a TV show increases memorization of the ad message. Memorization is an important lever marketers should consider when investing in advertisement campaigns. Indeed, if the advertisement can capture the individual attention, then memorization of the product or the brand is essential to create a connection with the consumer, induce familiarity and thus, increase the chances of product purchase. Besides cognitive outcomes, affective reactions of pleasure and arousal toward the advertisement are influenced by the media context. Positive emotions may impact the online word-of-mouth about the ads. Hence, advertisers and TV networks should carefully consider the emotions elicited by certain ads. Instead of automatically placing ads in the program breaks, they may allocate ads into different programs or in different positions in a certain program, depending on the cognitive and emotional reactions generated by that program. Clearly, this is applicable in pre-produced programs only, not in live events or reality shows where the content is not defined.

We should consider that the level of attention, memorization or affective reactions to be induced may vary among brands and products and depends on the marketing objectives a brand pursues. Certainly, campaigns need to be placed across different mediums and content to reach the target market, which is fundamental to growth (Graham and Kennedy, 2022) However, managers should carefully consider the media and the specific context in which they place their campaigns to reinforce (and even more important not undermine) the meanings and feelings they want to transmit with their messages. For instance, sportswear advertising usually wants to transmit energy, excitement, and power. Thus, placing sportswear advertisements inside exciting and arousing TV shows can reinforce the feelings companies want to convey. Above that, marketers should pay attention to the values characterizing television programs, as these are going to be attached to the brand advertised. The context should be chosen accordingly with the brand positioning and the brand image (above the channel target audience) to avoid confusion or a misalignment between the brand identity and the brand image. Investments in editorial content quality may, in this regard, positively affect advertising revenues, through an enhancement of the perceived quality of the brand. This is a key point for media companies, that can enhance their value proposition to brands looking for advertising space, by investing in the quality of their television content. This could be a desirable strategy to relaunch the effectiveness of television advertising as a way to spur a positive brand image. Above that, in light of research on affect transfer, the ad sequence inside the commercial breaks should be carefully managed. This serves to ensure that, above the right television content, the ads are placed along other commercials which do not undermine its value and of course, do not make consumers switch channel during the commercials break. This negative externality may indeed affect the network’s revenues (Shi et al., 2022).

## 9 Limitations and directions for future research

The study analyses six advertising messages about six consumer good brands, familiar to the population from which the sample has been selected and with a wide target market. The pre-screening test, assessing the attitude toward the brands, helps in avoiding possible distortions due to the specific brands proposed. However, different brands might potentially generate different reactions in consumers. For instance, new ones may generate a stronger halo effect as consumers do not have a prior well-defined attitude toward the brand. Familiar brands indeed have been associated with different neural activation compared to unfamiliar brands (Schaefer et al., 2006). Thus, further research should extend the study to new brands, unknown to the target market. In the same perspective, future studies may explore the positive versus negative brand contrast as research suggests consumer reactions may differ in the two scenarios.

Secondly, we displayed advertising messages of 30 s each. Shorter or longer messages (for instance 15 15-s advertisement) might result in a different halo effect. Moreover, communication messages displaying forms of interaction (such as a QR code) may generate different reactions, in terms of attention or engagement. Future research should explore if and how the halo effect manifests in different advertising typologies.

Thirdly, our aim is to measure the impact of the TV show exposure on the advertisement, thus all physiological measures were obtained by temporarily averaging their values during the vision of each content of the experiment (television program and advertisement). The mean values thus obtained were related to the mean value recorded during the vision of a neutral image (baseline). In this way, it was possible to evaluate the variation of the emotional state of the subject against the steady state. However, such approach does not allow to track significant variation in cognitive and emotional processing over time, due to context effects of television on advertising. Future research may explore the halo effect over time, for instance by analyzing the last 60 or 30 s of the television clip.

Finally, the current work investigates the effect of television content on advertisement responses. The reverse pattern could be an interesting area of exploration, i.e., the influence exerted by advertisements on television programs. Recent research displays interesting results on this relationship, showing that exposure to advertising messages before a television program increases attention toward product placement and reduces consumption enjoyment (Russell et al., 2017). Hence, further research may delve more into this area.

## Data availability statement

The raw data supporting the conclusions of this article will be made available by the authors, without undue reservation.

## Ethics statement

Ethical approval was not required for the studies involving humans because local legislation does not require it. There are several reasons why an explicit approval by an Ethics Committee was not necessary: the participants are adults, the research does not involve vulnerable subjects, the participation in the study is voluntary, it is a minimal risk research, all data collected is treated anonymously. The studies were conducted in accordance with the local legislation and institutional requirements. The participants provided their written informed consent to participate in this study.

## Author contributions

DB: Writing – original draft. GN: Writing – review & editing.
